# Supplementary material for: The rewiring of a terminal selector regulatory cascade generates convergent neuronal laterality
Source: PLoS Genet. 2026 Feb 11;22(2):e1011782. doi: 10.1371/journal.pgen.1011782 (PMC12919926; doi:10.1371/journal.pgen.1011782)
Supplement: S1 Table — (DOCX) [file pgen.1011782.s001.docx]

**S1 Table.** Nematode strains

| **Gene *(Ppa-)*** | **Allele** | **Strain** | **Genetic Lesion or Genotype** |
| --- | --- | --- | --- |
| *--* | *wild type* | PS312 | *P. pacificus* California reference strain |
| *gcy-22.3* | *csuEx90* | RLH334 | *Ppa-gcy-22.3p::GFP; Ppa-egl-20p::turboRFP* |
| *che-1* | *ot5012* | OH17879 | 4 bp insertion causing frameshift and STOP. 2x outcrossed. |
| *che-1* | *csuEx93* | RLH335 | *Ppa-che-1pei::optRCaMP; Ppa-che-1pei::optGFP; Ppa-egl-20p::turboRFP* |
| *gcy-22.1* | *csuEx74* | RLH375 | *gcy-22.1p::GFP; Ppa-egl-20p::RFP* |
| *gcy-22.2* | *csuEx104* | RLH376 | *gcy-22.2p::GFP; Ppa-egl-20p::RFP*  Heather on plasmid, co-injection marker |
| *gcy-22.4* | *csuEx102* | RLH377 | *gcy-22.4p::GFP; Ppa-egl-20p::RFP* |
| *gcy-22.5* | *csuEx105* | RLH378 | *gcy-22.5p::GFP; Ppa-egl-20p::RFP* |
| *gcy-5* | *csuEx84* | RLH379 | *gcy-5p::GFP; Ppa-egl-20p::RFP* |
| *gcy-7.2* | *csuEx108* | RLH380 | *gcy-7.2p::GFP; Ppa-egl-20p::RFP* |
| *gcy-8.1* | *csuEx101* | RLH381 | *gcy-8.1p::GFP; Ppa-egl-20p::RFP* |
| *gcy-8.2* | *csuEx100* | RLH382 | *gcy-8.2p::GFP; Ppa-egl-20p::RFP*  (no expression) |
| *das-1* | *csu222* | RLH307 | EMS-induced *die-1* allele. 2x outcrossed to wild type. |
| *das-2* | *csu223* | RLH383 | EMS-induced; uncloned |
| *die-1* | *csu225* | RLH346 | CRISPR/Cas9-induced *die-1* genocopy of *das-1(csu222).* 2x outcrossed to wild type. |
| *pash-1* | *csu227* | RLH344 | CRISPR/Cas9-induced substitution mutation. TCA(S)>TAC(Y); 2x outcrossed to wild type. |
| *ttx-1* | *csu150* | RLH253 | CRISPR/Cas9-induced 9 bp in-frame insertion. 2x outcrossed to wild type. |
| *cog-1-3’ UTR GFP sensor* | *csuEx106* | RLH353 | *Ppa-che-1pei::optGFP:cog-1-3’ UTR; Ppa-egl-20p::RFP* |
| *cog-1-3’ UTR mutant* | *csu239* | RLH369 | CRISPR/Cas9-induced substitution mutation in site A1. Modest coiler. |
| *cog-1-3’ UTR mutant* | *csu252* | RLH370 | CRISPR/Cas9-induced substitution mutations in site D. Non-coiler and non-*Egl.* |
| *cog-1-3’ UTR mutant* | *csu253* | RLH371 | CRISPR/Cas9-induced substitution mutations in sites A1 and D. Severe egg-laying defective (*Egl*). |
| *cog-1-3’ UTR mutant* | *csu254* | RLH372 | CRISPR/Cas9-induced substitution mutations in sites A1 and D. Non-coiler and non-*Egl.* |
| *cog-1-3’ UTR mutant* | *csu255* | RLH373 | CRISPR/Cas9-induced 337 bp deletion. Modest coiler. |
| *cog-1-3’ UTR mutant* | *csu256* | RLH374 | CRISPR/Cas9-induced substitution mutations in sites A1 and A2. Modest coiler. |
| *miR-8345-3p*  *mutant* | *csu259* | RLH390 | CRISPR/Cas9-induced 34 bp deletion in miR-8345 seed region |
| *miR-8345-3p*  *mutant* | *csu265* | RLH391 | CRISPR/Cas9-induced 12 bp deletion in miR-8345 non-seed region |
| *ttx-1(csu150); che-1(ot5012)* | *---* | RLH392 | Double mutant |
