# Supplementary material for: The rewiring of a terminal selector regulatory cascade generates convergent neuronal laterality
Source: PLoS Genet. 2026 Feb 11;22(2):e1011782. doi: 10.1371/journal.pgen.1011782 (PMC12919926; doi:10.1371/journal.pgen.1011782)
Supplement: S2 Table — (DOCX) [file pgen.1011782.s002.docx]

**S2 Table.** Plasmids

| **Reporter** | **Plasmid** | **Promoter length (bp)** |
| --- | --- | --- |
| *Ppa-egl-20p::optGFP:rpl-28-3’ UTR* | *pZH008* | NA |
| *Ppa-gcy-22.1p::optGFP* | *pHC29* | ~2 kb |
| *Ppa-gcy-22.2p::optGFP* | *pID10* | 980 bp |
| *Ppa-gcy-22.4p::optGFP* | *pID9* | 990 bp |
| *Ppa-gcy-22.5p::optGFP* | *pID11* | ~1.5 kb |
| *Ppa-gcy-5p:: optGFP* | *pVL1* | 1 kb |
| *Ppa-gcy-7.2p:: optGFP* | *pID4* | 695 bp |
| *Ppa-gcy-8.1p:: optGFP* | *pID16* | 970 bp (no expression) |
| *Ppa-gcy-8.2p:: optGFP* | *pID17* | ~1.5 kb (no expression) |
| *Ppa-che-1pei::optGFP:cog-1-3’ UTR* | *pDC18* | ~3 kb |

*”pei”:* promoter + first exon + first intron
