# Supplementary material for: The rewiring of a terminal selector regulatory cascade generates convergent neuronal laterality
Source: PLoS Genet. 2026 Feb 11;22(2):e1011782. doi: 10.1371/journal.pgen.1011782 (PMC12919926; doi:10.1371/journal.pgen.1011782)
Supplement: S3 Table — (DOCX) [file pgen.1011782.s003.docx]

**S3 Table.** Primer sequences

| **Gene (*Ppa-)*** | **Primer** | **Sequence (5’>3’)** |
| --- | --- | --- |
| *die-1* | RHL1728  (crRNA) | GTATCGATATTAGCAATCAC |
|  | RHL1705 | CTCAGAACACATATGCTCAAG |
|  | RHL1706 | AATGAACTAATCCATGAGATATC |
|  | RHL1707 | GTTCCCACTGGATACGATAC |
|  | RHL1709  (ssDNA) | ATGGATTACAGATGCATGGAATAGTTATCGATGAGAATAAACCAGTGATTACTAATATCGATACTCGAGAGAGGGAAAGAACGGGTGAACTGACATTCAG |
| *pash-1* | RHL1729  (crRNA) | AGGTGATTGAGTACTCAAAG |
|  | RHL1731 | CGTGATCAGCAACGTTTCT |
|  | RHL1732 | AAGATGCGTTCATTCGAAGGC |
|  | RHL1733 | CAACACTAGTTCGTACTGTTGA |
|  | RHL1730  (ssDNA) | GGGCTTTGACATGCTCGCAGTCGAGGATCCCAAGGTGATTGAGTACTTTAAGAGAACCGGCCGGCAGCTGCCTCACACCATTCTACTGGTATGATCTTTC |
| *ttx-1* | RHL1378 (crRNA) | TTGGCCGCAGAGCTGACCGA |
|  | RHL1379 | CCCACTTAGGTATTCATAATCCC |
|  | RHL1380 | TCCTTTAGGCTAGGCTAGGTAC |
|  | RHL1381 | CACGCAAGCAATGTTAGGCATG |
| *gcy-22.1p::GFP* | RHL1165 | ACACAAGCTCTACTGTTTATG (2 kb promoter) |
|  | RHL1171 | GAAAAATACAAATACTTAAAGG |
| *gcy-22.2p::GFP* | RHL1477 | GAGAGAAAGGACATTTTTATTTAC (~990 bp promoter) |
|  | RHL1478 | TGTTTGTCGGTTAACTGAAA |
| *gcy-22.4p::GFP* | RHL1480 | GTTTTGGTCACGTCCAAG (~990 bp promoter) |
|  | RHL1481 | AAATGTGAAAAGAAGAAACG |
| *gcy-22.5p::GFP* | RHL1483 | CATGGCAGCTTACTGTAG (~900 bp promoter) |
|  | RHL1484 | CACCGAAAGAGATGGCTGCG |
| *gcy-5p::GFP* | RHL1332 | AGTTTGAAACTCAAGAATTATTC (~1 kb promoter) |
|  | RHL1333 | CTGAAGTCACTCCATTAATATG |
| *gcy-7.2p::GFP* | RHL1557 | GGATCTGGAAGGATATGTATTGTAACTATAC (695 bp promoter) |
|  | RHL1558 | CTGCACTCCCAATCCAGCCG |
| *gcy-8.1pe::GFP* | RHL1677 | TGACGCATGATAACCCGACT (970 bp promoter + 98 bp exon 1) |
|  | RHL1676 | GTTGAGATCCTCGCGAGTGT |
| *gcy-8.2pe::GFP* | RHL1679 | ATCATAACGACACGTGCCGC (1516 bp promoter + 81 bp exon 1) |
|  | RHL1680 | TGTCGCTCTTTCGAGATCAC |
| *cog-1* | RHL1801  (crRNA1 in 3’ UTR) | TGAAAGGGCTGAATAAACTG |
|  | RHL1802  (crRNA2 in 3’ UTR) | GATAACAGGGCTACGGACAG |
|  | RHL1799 | TCGGAGCAATTAGCAGCATT |
|  | RHL1800 | TCCATTGACTTGTCCAGCGA |
|  | RHL1811 | TATATCAACTAGATTAGATCGTAG |
|  | RHL1844  (crRNA in coding) | TTCATCGTCGAGTCCTTGCG |
|  | RHL1803  (ssDNA) | CCATATTTCAAATACCGATCCATTTCCCGTAAGGGCTAGCTAGCATTCAGCCCTTTCATTCCTTTCTTTGACATCCCCTCCCTAATGATTGAGACAGTTTTACAGTTATGACACGTCCGCACAGAATTGAAGCAT |
|  | RHL1842 | GAACATAAGGATAAGGCTTTAAG |
|  | RHL1843 | GCAGTTGCTAGATTACATGTGAA |
| *miR-8345* | RHL1859  (crRNA) | TAGTAGAAATATCGACAGTG |
|  | RHL1856 | TAGAGTGAAGCCCTTCATCTA |
|  | RHL1858 | TTGGAAAGTCTTATCTCGGAA |
