# Supplementary material for: The rewiring of a terminal selector regulatory cascade generates convergent neuronal laterality
Source: PLoS Genet. 2026 Feb 11;22(2):e1011782. doi: 10.1371/journal.pgen.1011782 (PMC12919926; doi:10.1371/journal.pgen.1011782)
Supplement: S4 Table — (DOCX) [file pgen.1011782.s004.docx]

**S4 Table.** *in situ* HCR probes

| **Gene *(Ppa-)*** | **Transcript** | **Initiator (fluorophore)** | **Neuronal expression** | **Related Figure(s)** |
| --- | --- | --- | --- | --- |
| *gcy-22.1* | PPA34960 | B4 (AF 546) | ASER only | SI Fig. 1 |
| *gcy-22.2* | PPA34961 | B4 (AF 546) | ASER only | SI Fig. 1 |
| *gcy-22.3* | Contig12-snapTAU.506 | B2 (AF 488),  B4 (AF 546) | ASER only | Fig. 3; *SI Fig. 3-4* |
| *gcy-22.4* | PPA41119 | B4 (AF 546) | ASER only | SI Fig. 1 |
| *gcy-22.5* | PPA03763 | B2 (AF 488) | ASER only | Fig. 4 |
| *gcy-5* | PPA13334 | B4 (AF 546) | ASE (too weak) | SI Fig. 2 |
| *gcy-7.1* | PPA41433 | B4 (AF 546) | ASEL only | Fig. 3 |
| *gcy-7.2* | PPA02209 | B2 (AF 488),  B4 (AF 546) | ASEL only | Fig. 3 |
| *gcy-7.3* | PPA12710:  DN24356_c0_g1_i1; DN21395_c0_g1_i1 | B4 (AF 546) | ASEL only | Fig. 3 |
| *gcy-8.1* | PPA24212 | B4, B5 (AF 546, AF 647) | Both AFDs | Fig. 4 |
| *gcy-8.2* | PPA41407 | B5 (AF 647) | Both AFDs | Fig. 4 |
| *gcy-8.3* | PPA05923 | B5 (AF 647) | Both AFDs | Fig. 4 |
| *die-1* | PPA12810 | B5 (AF 647) | Many,  ASEL not ASER | SI Fig. 4 |

All probe sets are comprised of 30 pairs of oligos.
