# Supplementary figures and images for: The rewiring of a terminal selector regulatory cascade generates convergent neuronal laterality
Source: PLoS Genet. 2026 Feb 11;22(2):e1011782. doi: 10.1371/journal.pgen.1011782 (PMC12919926; doi:10.1371/journal.pgen.1011782)

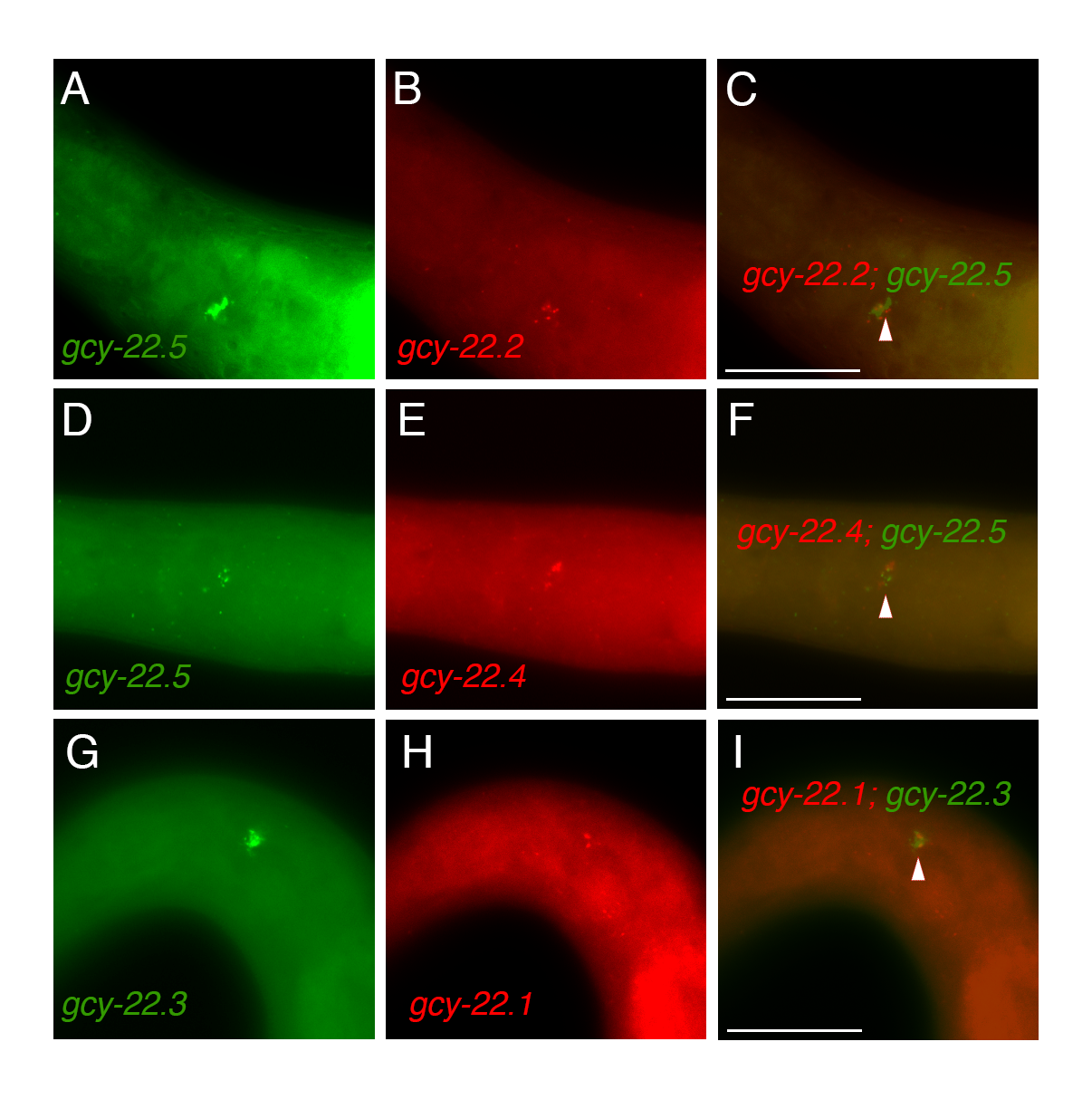

Supplement: S1 Fig — (A-C) gcy-22.2 co-localized with gcy-22.5 in a J4 hermaphrodite. (D-F) gcy-22.4 co-localized with gcy-22.5 in an adult hermaphrodite. (G-I) gcy-22.1 co-localized with gcy-22.3 in a J4 hermaphrodite. Triangles indicate co-expression of the gcy-22 paralogs. The scale bar represents 25 µm. (TIF) [file pgen.1011782.s005.tif]

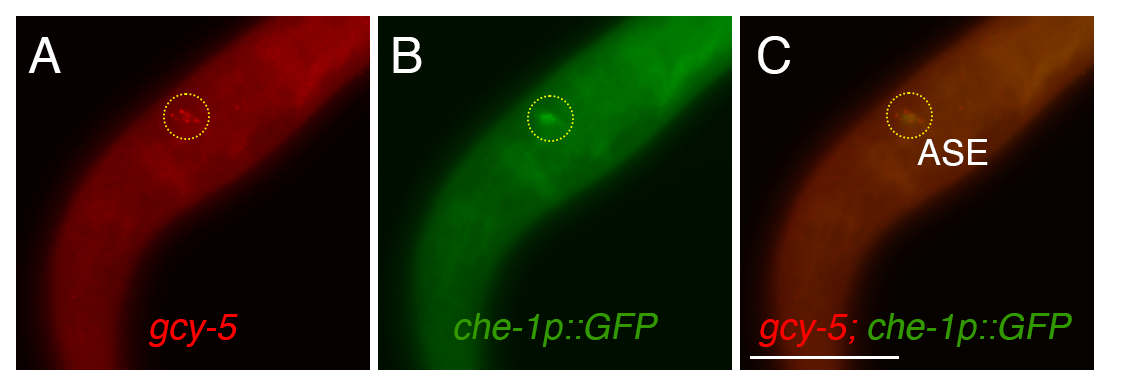

Supplement: S2 Fig — (A-C) A J3 hermaphrodite shows gcy-5 co-localization with the che-1p::GFP reporter in an ASE neuron based on cell body position (AFD would be more anterior). The scale bar in (C) represents 25 µm. (TIF) [file pgen.1011782.s006.tif]

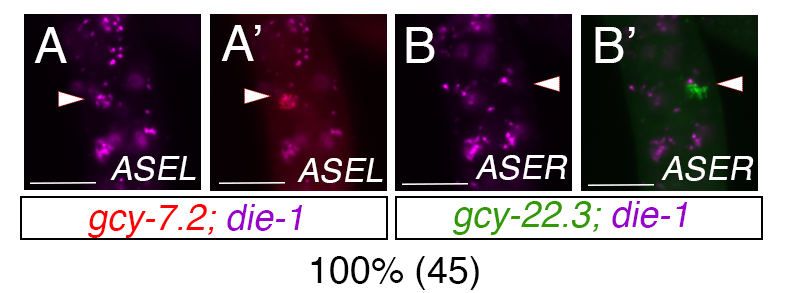

Supplement: S3 Fig — (A-A’) HCR FISH of a J3 larva shows die-1(B5) can co-express with gcy-7.2(B4) transcripts in the ASEL neurons. (B-B’) In contrast in the same animal, die-1(B5) do not co-express with gcy-22.3(B2) transcripts in the ASER neurons. Scale bar represents 5 µm. (TIF) [file pgen.1011782.s007.tif]

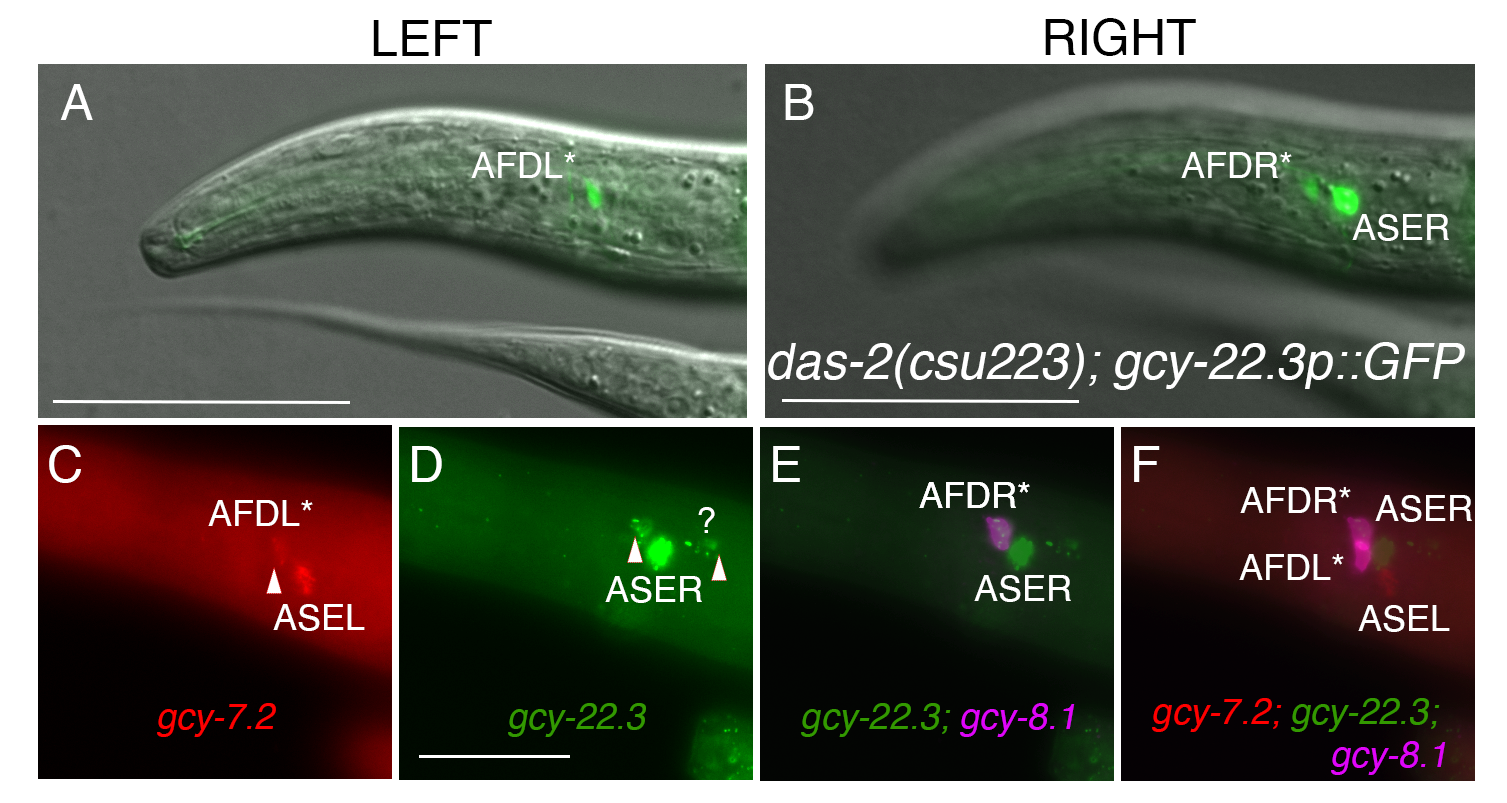

Supplement: S4 Fig — (A-B) Ectopic expression of the ASER gcy-22.3p::GFP reporter in AFD neurons in a J3 hermaphrodite. The scale bars represent 50 µm. (C-F) Ectopic expression of the gcy-7.2 and gcy-22.3 transcripts were found in AFD* neurons that also co-express the gcy-8.1 marker. “?” in (D) denotes an unidentified neuron posterior to the ASEs observed in 24% animals (12 out of 50). (F) Overlay image of all three channels (C-E). Triangles in (C) and (D) indicate neurons with ectopic expression of ASE markers in the AFD neurons. The scale bar in (D) represents 25 µm in panels C-F. (TIF) [file pgen.1011782.s008.tif]

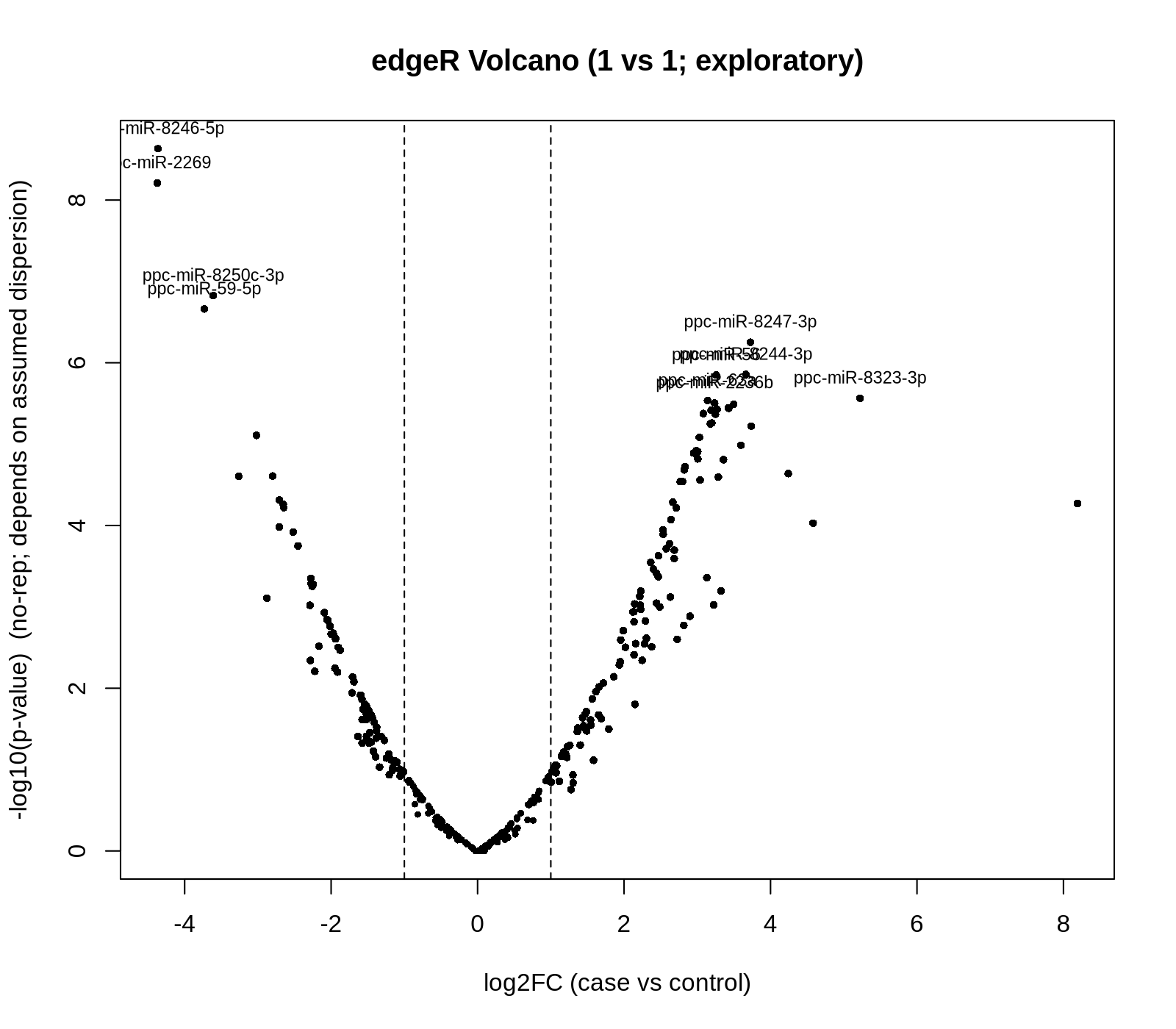

Supplement: S1 Appendix — Differential Expression list of hairpin miRNA with volcano plot (csv and png). Differential Expression list of mature miRNA with volcano plot (csv and png). Mirtrace length plot (png). Mirtrace phred plot (png). Mirtrace complexity plot (png). Mirtrace contamination plot. (ZIP) [file pgen.1011782.s013.zip › smRNAseq pash1/mature.volcano_noRep.png]

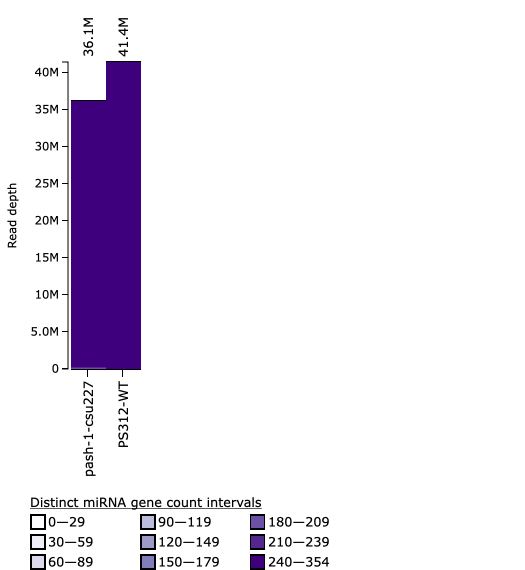

Supplement: S1 Appendix — Differential Expression list of hairpin miRNA with volcano plot (csv and png). Differential Expression list of mature miRNA with volcano plot (csv and png). Mirtrace length plot (png). Mirtrace phred plot (png). Mirtrace complexity plot (png). Mirtrace contamination plot. (ZIP) [file pgen.1011782.s013.zip › smRNAseq pash1/mirtrace-complexity-plot.png]

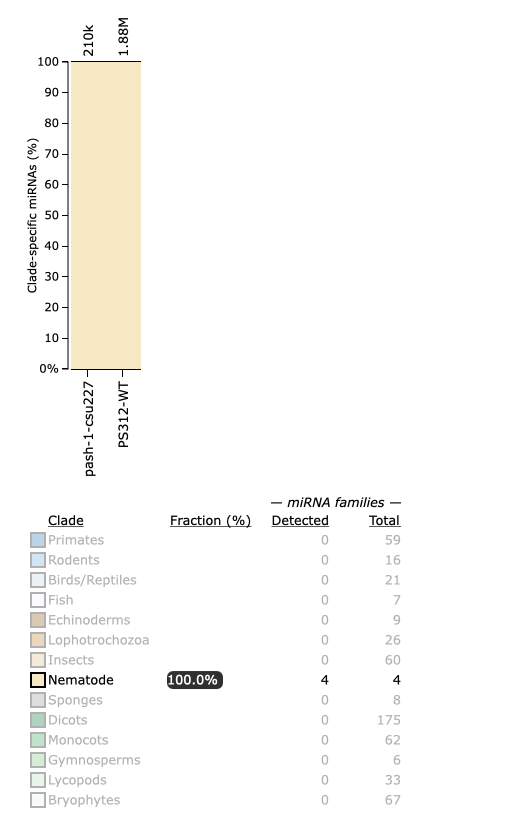

Supplement: S1 Appendix — Differential Expression list of hairpin miRNA with volcano plot (csv and png). Differential Expression list of mature miRNA with volcano plot (csv and png). Mirtrace length plot (png). Mirtrace phred plot (png). Mirtrace complexity plot (png). Mirtrace contamination plot. (ZIP) [file pgen.1011782.s013.zip › smRNAseq pash1/mirtrace-contamination-plot.png]

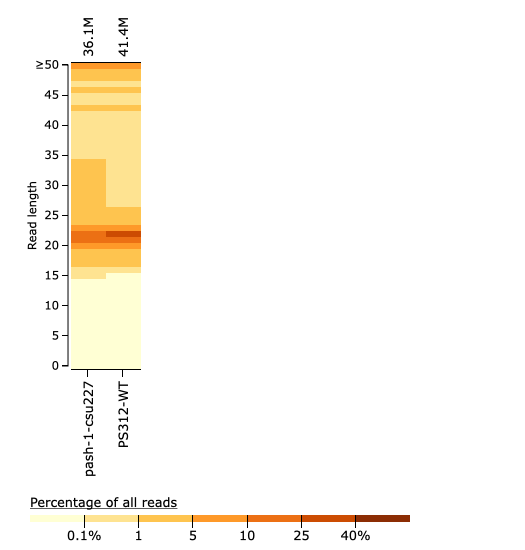

Supplement: S1 Appendix — Differential Expression list of hairpin miRNA with volcano plot (csv and png). Differential Expression list of mature miRNA with volcano plot (csv and png). Mirtrace length plot (png). Mirtrace phred plot (png). Mirtrace complexity plot (png). Mirtrace contamination plot. (ZIP) [file pgen.1011782.s013.zip › smRNAseq pash1/mirtrace-length-plot.png]

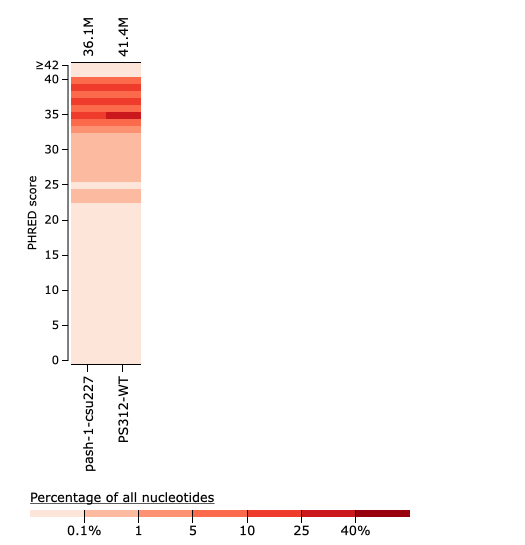

Supplement: S1 Appendix — Differential Expression list of hairpin miRNA with volcano plot (csv and png). Differential Expression list of mature miRNA with volcano plot (csv and png). Mirtrace length plot (png). Mirtrace phred plot (png). Mirtrace complexity plot (png). Mirtrace contamination plot. (ZIP) [file pgen.1011782.s013.zip › smRNAseq pash1/mirtrace-phred-plot.png]

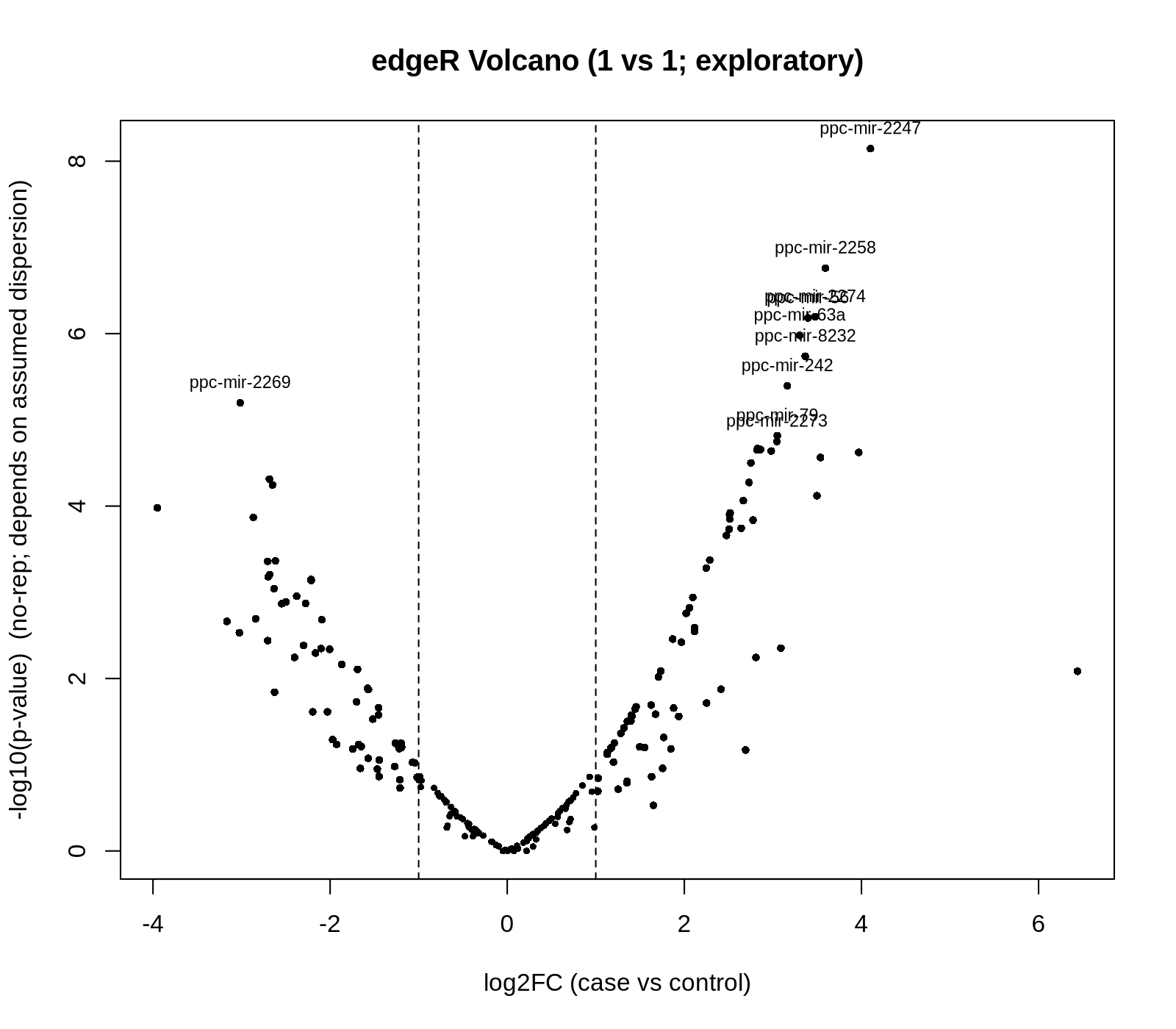

Supplement: S1 Appendix — Differential Expression list of hairpin miRNA with volcano plot (csv and png). Differential Expression list of mature miRNA with volcano plot (csv and png). Mirtrace length plot (png). Mirtrace phred plot (png). Mirtrace complexity plot (png). Mirtrace contamination plot. (ZIP) [file pgen.1011782.s013.zip › smRNAseq pash1/hairpin.volcano_noRep.png]
