# Supplementary material for: The rewiring of a terminal selector regulatory cascade generates convergent neuronal laterality
Source: PLoS Genet. 2026 Feb 11;22(2):e1011782. doi: 10.1371/journal.pgen.1011782 (PMC12919926; doi:10.1371/journal.pgen.1011782)
Supplement: S6 Fig — DNA alignment of the CRISPR/Cas9-generated P. pacificus die-1, pash-1, ttx-1, and miR-8345 mutant alleles and their predicted amino acid changes. The GCT > ACT (A359T) substitution of die-1(csu222, csu225) is predicted to affect the sixth zinc-finger domain. The TCA > TAC (S494Y) mutation of Ppa-pash-1(csu227) is likely to affect an RNA-binding domain, dRBD2. The in-frame 9 nucleotide complex insertion in Ppa-ttx-1(csu150) results in a “CVIW” replacement of the V residue near the N-terminus of the protein. Bold black letters indicate mutations. For miR-8345 alleles csu259 and csu265, magenta highlights the mature miRNA and bold sequences indicate the seed region. (DOCX) [file pgen.1011782.s010.docx]

*Ppa-die-1*(PPA12810): ATGAGAATAAACCAGTGATTGCTAATATCGATACTCGAGAG

*csu225* (G>A): ATGAGAATAAACCAGTGATT**A**CTAATATCGATACTCGAGAG

*Ppa-*DIE-1(PPA12810): MHGIVIDENKPVIANIDTRERERTGELTFR 375

*csu225* (A->T): MHGIVIDENKPVI**T**NIDTRERERTGELTFR 375

*Ppa-pash-1*(PPA05475): ATCCCAAGGTGATTGAGTACTTTAAGAGAACCGGCCGGCAGCT

*csu227* (2 bp sub): ATCCCAAGGTGATTGAGTACT**CA**AAGAGGACCGGCCGGCAGCT

*Ppa-*PASH-1(PPA05475): DTMEGFDMLAVEDPKVIEYSKRTGRQLPHTILL 507

*csu227*(S->Y): DTMEGFDMLAVEDPKVIEY**Y**KRTGRQLPHTILL 507

*Ppa-ttx-1*(PPA26714): CGTACCTGCCCTCGGTCAGC

*csu150* (+9 bp): CGTACCTGCCCTCG**TGC**GTC**ATCTGG**AGC

*Ppa-*TTX-1(PPA26714): DAPIFSDMTYLPS-V--SSAANMSHVAASMYSGK 59

*csu150* (+CVIW): DAPIFSDMTYLPS**CVIW**SSAANMSHVAASMYSGK 62

*Ppa-cog-1*(PPA04715): AGCTCCCCTCAGCCGCGCAAGGACTCGACGATGAA

csu257(-8 bp): AGCTCCCCTCAGCCGCG--------CGACGATGAA

csu258(A>T, +45 bp): AGCTCCCCTCAGCCGCGC**T**AG**[]GA**CTCGACGATGAA

*Ppa-cog-1*(PPA04715): EVDTVQLTPSPEPSSPQPRKDSTMKTGSYSISNLLEKKEE 68

csu257: EVDTVQLTPSPEPSSPQPRDDEDWQLLHFKSA* 60

csu258: EVDTVQLTPSPEPSSPQPR* 47

*ppc-miR-8345-3p*: CAAATAGTGG**TTTTGTAT**GACCTCACTGTCGATATTTCTACTAA

*csu259* (-34 nt): ------------------------------- ATATTTCTACTAA

*csu265* (-12 nt): CAAATAGTGG**TTTTGTAT**GACCTCACT------------ACTAA
