# Supplementary material for: The rewiring of a terminal selector regulatory cascade generates convergent neuronal laterality
Source: PLoS Genet. 2026 Feb 11;22(2):e1011782. doi: 10.1371/journal.pgen.1011782 (PMC12919926; doi:10.1371/journal.pgen.1011782)
Supplement: S8 Fig — (DOCX) [file pgen.1011782.s012.docx]

>ppc-miR-2251b-3p MIMAT0032849

UAACUGGGAUUCUGUGGAGAUCU

>ppc-miR-81 MIMAT0011656

UGAGAUCAUAGUGAAAGCUACU

>ppc-miR-8353-3p MIMAT0033267

GAAAUGAGUCUGACUUGUAUGA

>ppc-miR-8345-3p MIMAT0033237

UUUUGUAUGACCUCACUGUCGAUA

>ppc-miR-8364f-3p MIMAT0033110

AGACAGUGUGGAAAAUGAAGAGA
