# Supplementary material for: The rewiring of a terminal selector regulatory cascade generates convergent neuronal laterality
Source: PLoS Genet. 2026 Feb 11;22(2):e1011782. doi: 10.1371/journal.pgen.1011782 (PMC12919926; doi:10.1371/journal.pgen.1011782)
Supplement: Attachment — Submitted filename: PGENETICS_Responses to reviewers.pdf [file pgen.1011782.s014.pdf]

PGENETICS-D-25-00733
The rewiring of a terminal selector regulatory cascade generates convergent neuronal laterality

PLOS Genetics

Dear Dr. Hong,

Thank you for submitting your manuscript to PLOS Genetics. After careful consideration, we feel that it has merit but does not fully meet PLOS Genetics's publication criteria as it currently stands. Therefore, we invite you to submit a revised version of the manuscript that addresses the points raised during the review process.

Please submit your revised manuscript within 60 days Sep 27 2025 11:59PM. If you will need more time than this to complete your revisions, please reply to this message or contact the journal office at plosgenetics@plos.org. When you're ready to submit your revision, log on to https://www.editorialmanager.com/pgenetics/ and select the 'Submissions Needing Revision' folder to locate your manuscript file.

Please include the following items when submitting your revised manuscript:

* A rebuttal letter that responds to each point raised by the editor and reviewer(s). You should upload this letter as a separate file labeled 'Response to Reviewers'. This file does not need to include responses to any formatting updates and technical items listed in the 'Journal Requirements' section below.

Authors' summary on the revised manuscript

We thank the reviewers for taking their time to give thoughtful comments and suggestions to improve our manuscript. In our revised manuscript, to address the major concern regarding support for actual miRNA involvement in the regulation of *cog-1,* we included fresh experimental results of mutations in *miR-8345*, a negative regulator of *cog-1* with two conserved putative binding sites in the *cog-1* 3' UTR. We found a significant majority of *miR-8345* mutant animals show a complete conversion of the ASEL neuron into the ASER fate (2xASER), with a much lower frequency of having hybrid ASELs than in the *cog-1(csu255)* 3' UTR allele with the large deletion. We also engineered loss-of-function *cog-1* alleles in the coding region to provide additional support for the claim that reduction in COG-1 function is responsible for the misexpression of the terminal effector *gcy-7.2*. Specifically, we show that *cog-1* loss-of-function alleles in the coding region (*csu257, csu258*) and the *cog-1(csu253)* 3' UTR mutant share the hybrid 2xASEL* phenotype, and thus the 3' UTR contains positive regulatory regions as well. We also found a complete lack of *gcy-8.1* expression in the AFD neurons in the newly generated *ttx-1; che-1* double mutant, suggesting that ttx-1 also has a positive role in regulating AFD-specific *gcy* expression. The original text was shortened and specific figure panels were cited to

enhance clarity. We updated the summary figure on the current findings, which we believe represent an advancement for understanding the evolution of developmental neurobiology.

Reviewers' comments:

Reviewer's Responses to Questions

Comments to the Authors:
Please note here if the review is uploaded as an attachment.

Reviewer #1: In this manuscript the authors use the comparison between C. elegans and P. pacificus as a way to explore how gene regulatory evolution can be linked to phenotype, specifically in the development of the nervous system. The authors make a number of findings about how gene expression regulation is implicated in the development of asymmetry in the P. pacificus nervous system. The work is clear and most of the experiments are performed well. I think one would have to say that the conclusions are of specialised interest and from only one species, it is difficult to make generalisations about how regulatory systems evolve. However, detailed exploration of neuronal asymmetry in emerging model organisms will be important in moving beyond studies in a few widely separated systems (ie C. elegans, Drosophila, mouse etc). Collecting data from different systems is clearly the first, necessary, step in this process so the present work is valuable. I have only two major criticisms of the manuscript which relates to the section on miRNAs:
1) Crucially, the authors conclusions are based in part on analysis of a Pash1 allele, which they have engineered on the basis of the well known ts allele in C. elegans. However, it does not seem clear to me how they have validated that this allele has the same effect on miRNA processing as in C. elegans. Moreover, the exact conditions they used for the permissive and non-permissive temperatures are not described in the results section and this should be added. Some validation that the allele behaves as the C. elegans allele, by high-throughput sequencing of small non-coding RNAs after shifting to the non-permissive temperature would be very useful here- it would also give insight into which miRNAs are possible candidates as, at least in C. elegans, not all miRNAs are responsive to pash-1 depletion. Small non-coding RNA sequencing is fairly straightforward now and can be outsourced to many companies without incurring huge costs. Combining this with mRNAseq analysis might further identify changes in gene expression resulting from miRNA depletion that could be linked to the neuronal phenotype (for example if one of the affected genes were cog-1 this would be consistent with the authors’ hypothesis that this is regulated by miRNAs).

To characterize potential temperature-sensitive phenotypes of *Ppa-pash-1(csu227)*, we looked for Mig, Dpy, and Egl phenotypes at 15°C and 20°C. However, we found that the frequency of Mig and Dpy in *pash*-1 mutant is not temperature dependent, and the *pash*-1 mutant does not show a higher Egl phenotype rate compared to the wildtype. Hence, the characterization of small RNA species using *Ppa-pash-1(csu227)* may not be as straightforward as in *C. elegans*. We now added the phenotype frequency of *Ppa-pash-*

1(csu227) at 15C vs 20C in SI Figure 5. We also acknowledge in the Discussion that the effects of the *pash-1* mutation on the specific miRNA implicated in the conserved binding sites are currently unknown. Since small non-coding RNAs appear to be quite divergent in *P. pacificus* (Sharma et al, BioRxiv 2024), we hope the current study would provide us with the basis for future funding support for properly characterizing the small non-coding RNAs of *Ppa-pash-1(csu227)* by RNAseq.

2) Similarly, in the second part of this section, the conclusion of which miRNAs are involved is incomplete because mutating the putative target regions in the 3'UTR could have other effects on the transcript besides interfering with miRNA regulation. The correct way to do these experiments is to alter the target sequence by adjusting one nucleotide within the seed target region, and then if there is a phenotype, attempting to supress the phenotype by making the complementary mutation in the hypothesized miRNA's seed sequence. This should then bring back the targeting and restore the phenotype.

We understand the reviewers concern that definitive proof of specific miRNAs involved in the regulation of *cog-1* 3'UTR requires the construction of compensatory mutations between individual candidate miRNA and each binding site. On the basis that a convincing *P. pacificus lsy-6*-like miRNA was initially lacking and that this was the first study of neuronal patterning in *P. pacificus*, our priority was to characterize the *Ppa-cog-1* 3' UTR as a regulatory nexus by identifying likely miRNA binding sites among the many conserved regions to make targeted mutations. Because phylogenetic footprinting identified at least 4 strongly conserved miRNA binding sites and subsequent targeted mutagenesis of the 3'UTR suggests at least 3 modes of post-transcriptional regulation, we hesitated initially to extend our investigation to include determination of these individual miRNAs since it would be beyond the scope of this work. In the revised manuscript, we show that *miR-8345* is very likely the *lsy-6* homolog acting on the 3'UTR: two *miR-8345* alleles mutants resulted in a strong 2xASER phenotype, which overlaps with the phenotype spectrum of the *cog-1(csu255)* 3'UTR deletion mutant.

There were a few minor errors in the text that I noticed, possibly some others, so it might be worth reviewing this carefully before any resubmission. One in particular is the following sentence:
"we found 1-many homologs of gcy-7 (ASEL-type) and gcy-22 156 (ASER-type) are present in the P. pacificus genome"
Do they mean 'many homologues' or '1-to-many' homologues?

Thank you for pointing this out. We meant '1-to-many' and this has been corrected in the text.

Reviewer #2: In this article, Castro et al. analyzed the evolution of a gene regulatory network regulating neuron type differentiation in nematodes. Nematodes are good model organisms to study neuronal cell type evolution as they offer a single neuron resolution. More precisely, they compared the network regulating the specification of the

left and right ASE neurons between Pristionchus pacificus and C. elegans. In C. elegans, the pair of ASE neurons displays molecular and functional left-right asymmetries and the gene regulatory network that regulates this asymmetry has been well characterized. In P. pacificus, the authors have previously established that ASE left and right are functionally different, however how this asymmetry is regulated remained to be established. In this article, using HCR-FISH, a genetic screen, CRISPR genome engineering and behavioral tests, the authors characterized the gene network regulating the differentiation of the ASE left and right neurons in P. pacificus. They observed both common features as well as differences with the C. elegans network. Overall, the results are interesting and convincing. However, the presentation of the results is sometimes a bit confusing and the manuscript could be improved before publication.

Major point:
The authors examine the role of cog-1 3'UTR using CRIPR mutations and looking at the effect on terminal marker expression (gcy-7.2, gcy-22.3, gcy-8.1) by HCR-FISH. This is a great approach to characterize the phenotypic consequences. However, the interpretation, in terms of molecular mechanism, is complicated by the fact that they did not analyze the direct consequences of these cog-1 3'UTR mutations on COG-1 protein production in cis. This could be done, for example, by using their Ppa-che-1p::gfp:cog-1 3' UTR transgene strategy. They have shown that this construct drives left-right asymmetric GFP expression in ASE. They could introduce their cog-1 3'UTR mutations in this construct and analyze the consequences on GFP expression.

We understand the concern regarding more evidence for direct consequences of the 3' UTR mutations on *Ppa-cog-1* transcript level using the transgenic *cog-1* 3' UTR sensor. However, stable transgenic strains with extrachromosomal arrays are ~100x more difficult to obtain in *P. pacificus* than in *C. elegans* (see Methods), in part due to transgene toxicity, while still encumbered by mosaicism and variable copy numbers. Therefore, we could not accomplish the making of new transgenic sensor strains for this revision. Rather, to show that a loss COG-1 function can phenocopy a mutant class in the *cog-1* 3' UTR, we knocked down *cog-1* with frameshift mutations in second exon that introduced early stop codons (there does not appear to be a cryptic second ATG in *Ppa-cog-1* as is found in the *Cel cog-1*). These putative *cog-1* null alleles (*csu257, csu258*) show 48-50% 2xASEL* hybrid phenotype resembling the *cog-1(csu253)* allele with mutations in the A1 and D binding sites of the 3' UTR (Table 7).

Minor points:
- In fig. 1, Ppa-gcy-7.1, Ppa-gcy-7.2 and Ppa-gcy-7.3 do not cluster with Cel-gcy-7. Could the authors explain why they consider these P. pacificus genes as orthologs of C. elegans gcy-7?

There are 4 ASEL-expressing rGCs in *C. elegans* known as the "*gcy-7* subgroup" (*gcy-6, gcy-7, gcy-14, gcy-20*)(Ortiz et al 2006), compared to 3 ASEL-expressing rGCs in *P. pacificus* (*gcy-7.1, gcy-7.2, gcy-7.3*). Since the *gcy-7* subgroup members between the closely related *C. briggsae*, and *C. elegans* already lacked 1-1 homology (for *gcy-6,*

gcy-14, gcy-20)(Ortiz et al 2006; Hong *et al* 2019), it seems to us most appropriate to assign the *P. pacificus* ASEL rGCs *gcy-7* members to the most-utilized terminal effector marker for ASEL fate, *gcy-7*. Therefore, although the non-lateralized *Cel gcy-13* homologs clustered the closest to the *P. pacificus gcy-7.1, gcy-7.2, gcy-7.3* paralogs, these *P. pacificus* ASEL-expressing rGC members are closer to the *C. elegans* "*gcy-7* subgroup" than the ASER-expressing "*gcy-5* subgroup".

- p9 lines 233-235: "The degree of reduction of gcy-8 expression in che-1 mutant animals is likely an underestimation, since only animals with at least one AFD neuron staining were scored but those without any AFD expression were not included in the overall count." Could the authors explain why those without any AFD expression were not included in the overall count.

As with all the probes we used in HCR staining, we found that the most individual samples do not stain at all so in order to control for loss of expression due to technical limitations (*i.e.* samples were differently permeable to FISH) versus a complete loss of endogenous gene expression due to changes in gene regulation, we scored only those samples showing some staining in the *gcy-8* paralogs.

- p10 lines 259-269: paragraph starting with "We next ask if the other gcy-7 and gcy-22 paralogs …". I find this paragraph a bit hard to follow, especially because the data are not shown. Showing data or adding more explanations could help.

We concur that this paragraph seems extraneous and have removed it.

- p14 lines 376-379: "Given the wildtype-like expression of gcy-8.1 and finger morphology in ttx-1 mutants, the primary role of ttx-1 in P. pacificus amphid neurons appears to be the repression of che-1-dependent ASE differentiation in the AFD rather than the promotion of AFD-specific fates." This seems to me an overstatement as the ttx-1 mutant used is a hypomorph and not a null.

Because a *ttx-1* null allele also doesn't exist in *C. elegans* due to embryonic lethality, we are comparing a similarly hypomorphic *Ppa-ttx-1(csu150)* allele to the reference hypomorph *Cel-ttx-1(p767)*. Although both hypomorphs share thermotaxis and daf-c phenotypes, *Ppa-ttx-1(csu150)* showed no observable change in *gcy-8* expression and AFD finger morphology.

To address the potential that *Ppa-ttx-1* also has a role in activating AFD-specific gcy genes in a *che-1* dependent manner, we generated *ttx-1(csu150); che-1(ot5012)* double mutants and observed their loss of all AFD (*gcy-8.1*) and ASE (*gcy-22.3* and *gcy-7.2*) expression (scoring only those worms showing expression of the independent intestinal gene marker, *Ppa-hsd-2*, for probe penetrance). This complete loss of *gcy-8.1* expression is more severe than in the *che-1* mutant alone (Table 2, 0% vs 28%

staining) and suggests *ttx-1* may also have positive, instructional role in AFD terminal differentiation. This dual function of TTX-1 in AFD differentiation is now addressed.

A *Ppa-ttx-1* null allele limited to the post-embryonic AFD neurons could potentially address this concern in the future (e.g. via a binding site mutation in the *ttx-1* cis-regulatory region).

- p15 lines 410-411 : “We found that Ppa-pash-1(csu227) mutants express two gcy-22.3p::gfp (ASER) neurons (Fig. 10).” Fig. 10 does not show gcy-22.3p::gfp expression but HCR-FISH.

We have removed “::gfp”

- p15 line 422: “Since in C. elegans lsy-6 miRNA repression of cog-1 is necessary for the ASER fate in the ASEL neuron” should be “Since in C. elegans lsy-6 miRNA repression of cog-1 is necessary for the ASER fate repression in the ASEL neuron”.

We have inserted “repression”

- p17 line 476: “the repression of ASER fate in the ASEL through binding site A1”. I do not see the evidence that this is via A1.

This sentence is now corrected to read “the repression of ASEL fate in the AFD neurons throughout the 337 bp region (mainly through site C)” to reflect the newly obtained miR-8345 result that showed the 2xASER phenotype.

- Legend of fig. 3: n numbers are missing. The authors could also refer the readers to table 1.

We have now inserted the n numbers and referred to Table 1 in the Figure 3 legend.

- Fig. 5: for C and D pictures the authors should also show the gcy-7.2 and gcy-22.3 signal without the gcy-8.1 signal (as they did with A and B).

We added 2 panels showing gcy-7.2 and gcy-22.3 staining alone, without gcy-8.1, for the same wildtype J1 sample.

- Fig. 8C: I guess “DL” is “Dauer larvae”, this should be clarified. If this is the case then the y axis label “Dauer Formation (%)” is not correct and should be “Percentage of animals”.

“DL” has been replaced with “Dauer” and the Y axis is “Percentage of animals.”

- Table 3: Wildtype embryos should be added for comparison.

We have included the wildtype embryo data from Table 4 in Table 3.

- SI Fig. 4: It seems that pictures A and B have been inverted.

Thank you for catching this oversight, we have now switched the images between panels A and B.

Reviewer #3: In this manuscript, the authors dissect how asymmetry in neuronal fates is established in the satellite species P. pacificus, which allow single cell homology comparison with C. elegans, and easy gene orthology assignments. They identify how miRNAs regulate lateralised expression of cog-1, and from there lateralised expression of gcy-22 and -8 family genes.

Overall, the experiments are rational, and properly presented, although I would appreciate more details on a few points. I feel however that the main weakness of the manuscript is in the presentation and interpretation of the results.
For example, the big point here is a comparison of gene regulation between C. elegans and P. pacificus; the regulatory model for P. pacificus is nicely laid out in figure 12. I would like to see the model fo C. elegans presented side by side, to allow readers to easily compare the two.

Thank you for this request. We have added the C. elegans regulatory model for ASE specification adjacent to the proposed P. pacificus model in Figure 13.

Another main point in the title is that this asymmetric expression is convergent between C. elegans and P. pacificus. However, the fact that the regulation is different does not imply that this asymmetry evolved independently - nowhere do the authors present evidence that the ancestral species had symmetrical fate assignment for ASEL/R. A more likely possibility is that this asymmetry is ancestral (since the same nervous system seems to be highly conserved among rhabditina, and this asymmetry is but the underlying mechanisms drifted over evolutionary timescales. This difference is not trivial; mechanistically, the latter case (also termed developmental systems drift) is more interesting, since it implies regulatory changes that maintain functionality throughout, for example through redundancy. The exaptation of ASE fate specification mechanisms for AFD fate, on the other hand, seems to fundamentally differ from C. elegans.
Right now, the discussion does not really put the results in a broader context; I would appreciate a discussion on this most salient point – i.e., developmental systems drift, here illustrated by a careful dissection of gene regulation in P. pacificus.
Incidentally, it might be worth if the authors insist on the evolutionary distance between

elegans and pacificus - which is larger than that with C. briggsae, on which most mechanistic comparisons with C. elegans rely.

We appreciate the suggestion to re-frame our current findings not as a case of clear convergence, due to the lack of evidence from an ancestral species basal to both Caenorhabditis and Pristionchus genera (i.e. in a Poikilolaimus sp.), but rather as an example of developmental systems drift (DSD). If we consider lateral asymmetry in the ASE neurons as a conserved trait, then the co-option of the bi-stable loop between die-1 and cog-1 in the regulation of AFD fate could be an exaptation of ASE fate specification. We have shown in this revised manuscript the involvement of miR-8345 functioning as a lsy-6 homolog (although their genomic locations are not syntenic), which has extended a minor but noticeable regulation to the AFD neurons. Thus, we can make a convincing case for understanding the precise underlying mechanism for how the cog-1 3' UTR mediates the regulation of AFD fate in the context of DSD.

A few more specific points:

the authors identified putative homologs via blastx search in wormbase. While this is perfectly fine as a first approach, this should be repeated with a dedicated sofware package such as Orthofinder – incidentally, if run with the genomes of other Pristionchus species, this could give information on how recent the duplications observed for gcy-7 and -22 are. Increasing the number of species can also improve the accuracy of gene trees.

Admittedly, we did move beyond BLASTX and WormBase search because the limitation was our ability to accurately ascertain endogenous gene expression for the gcy members to confirm a possible correlation between gene sequence similarity and neuronal expression. Since even ASEL/ASER-specific *gcy* homologs (e.g. *gcy-4, gcy-19*) within Caenorhabditis species showed significant evolutionary divergence in gene expression (Ortiz et al, Genetics, 2006), it was not surprising to find a lack of 1-1 orthology between *gcy* genes between *C. elegans* and *P. pacificus*. When we initiated this study 3 years ago, the number of complete genomes from other Pristionchus species was more limited, until recently (Yoshida et al, 2024). We hope to use Orthofinder for characterizing the evolution of *gcy* family members in other *Pristionchus* species in the future.

In section "Multiple miRNAs are implicated in regulation of ASE and AFD fates", the authors introduced a putative TS mutation in Ppa-pash-1. I would expect this mutation to be highly pleiotropic, disrupting many regulatory pathways. I am therefore wondering whether proper cell identity can be ascertained. How healthy are these mutants? How would the authors expect them to respond to the chemotaxis assay used on die-1 mutants?

The *pash-1(csu227)* alleles is strongly pleiotropic for various developmental phenotypes, including Mig (gonad migration) and Dpy (dumpy) and are very slow

growing compared to *die-1(csu225)*. Therefore, it was not possible to perform chemotaxis behavioral assays on *Ppa-pash-1* mutants.

Later on in the same section, the authors identify multiple miRNAs binding in the 3' UTR of cog-1: mir-2251b, mir-81, mir-8353, mir-8345, mir-8364f, and identify 3 cis regulatory regions working co-operatively to drive proper expression of cog-1. In contrast, in C. elegans, only lsy-6 is described as regulating cog-1. However, there could be minor contribution from other miRNAs; have the authors checked whether there is any match for those found to be regulating cog-1 in Ppa?

We certainly agree that the conserved, often repeated 3' UTR sequence motifs with strong complementarity to identified mature *P. pacificus* miRNAs could represent multiple miRNA binding sites. We have identified C1 and C2 to be the most likely binding sites for miR-8345, which has a mutant phenotype resembling the pash-1 phenotype (loss of miRNA processing)(See below).

Minor points:

Line 402: "since it is unclear if a functional lsy-6 mRNA homolog exists in Pristionchus…" What exactly does that mean? No clear homolog? What are the closest matches? Do you know if lsy-6 is conserved beyond the Caenorhabditis genus?

The seed sequence of miR-8345-3p has the best match to *Cel-lsy-6* (Ahmed et al, 2013). However, Oliver Hobert's lab who is acquainted with *lsy-6* was doubtful of this homology because compared to the *lsy-6* secondary structure, the *miR-8345-3p* and *-5p* regions show poor complementarity with each other and is predicted to form a very tight hairpin that would be poorly processed by PASHA/DGCR8 (see attached). Thus, *lsy-6* was considered likely not to be conserved outside of the Caenorhabditis genus. However, in response to reviewers' comments we have generated two alleles of *miR-8345-3p* (*csu259, csu265*) with deletions in the seed regions and found both exhibited the 2xASER mutant phenotype strongly resembling the *pash-1* (lf) mutant. We have now included this new result in the revised manuscript.

Structure

```
c    -              A   U          a    aaaaa
 cauc AAAUGCGUCU GUA CAAAAUc ugu        c
 |||| |||||||||| ||| ||||||| |||
 guAG UUUACGCAGA UAU GUUUUag aca        u
a    C          G   -          -    aaaug
```

lsy-6

Structure

```
--  UU      --    U    U   u  uc
  UC  CAGUGA  UCAU CAAA CCa cu  a
  ||  ||||||  |||| |||| ||| ||
  AG  GUCACU  AGUA GUUU ggu ga  a
AU  CU      CC    U    U   -  ua
```

miR-8345

Line 289-290: it appears that gcy-22.3 and -22.5 are quite distant based on the tree in fig 1 – it is not at all unexpected that their regulation changed.

What was unexpected was that two *gcy* genes normally expressed asymmetrically in one ASE neuron would be subjected to both *die-1*-dependent and *die-1*-independent regulation. In *C. elegans*, there was no documentation of distant ASER-*gcy* genes on separate chromosomes (e.g. *gcy-5* subfamily and *gcy*-22) showing divergent regulation by *die-1.* To our knowledge the only similar example is of tax-2/tax-4 mutants showing ectopic *lim-6* expression (a homeobox TF normally expressed in ASEL) but not *gcy-7*. It may be that differential *gcy* gene regulation by *die-1* and *cog-1* exist in *C. elegans* but was not documented.

Line 813-814: Have the authors compared microsynteny between C. elegans and the two putative gcy-5 paralogs? It might be more accurate than chromosome assignment.

We checked the gene annotations around PPA02210_IV and PPA13334_II but neither paralog shares an obvious neighborhood of genes homologous those flanking Cel gcy-5.

Figure 7: violin plots should be replaced by scatterplots, which are more representative of the actual distribution and easier to read.

We have replaced the violin plots with scatterplots with individual data points.

Overall, the manuscript would benefit from a read-through to catch stylistic (eg. repetition of that in lines 64-65) or grammatical (eg. missing “to” at line 238) errors.

Thank you, we have amended these two specific errors.

Reviewer #4: This study is important because it provides insight into the role of terminal selectors and microRNAs in the development and evolution of neuronal asymmetry. Contrary to what was assumed in the field, the paper establishes that ASE neurons of the nematode P. pacificus display asymmetrical effector gene expression. The authors dissect a miRNA-associated gene regulatory network important for ASEL/ASER and AFD identity, which is suggested to have convergently evolved relative to C. elegans. Major strengths of this work include (a) use of HCR for reliable characterization of endogenous gene expression, (b) use of both unbiased (genetic screen) and biased approaches to identify key factors acting within the novel regulatory network, (c) the use of alternative approaches to determine the importance of miRNA regulation within the evolving network despite challenges associated with miRNA homology assignment, (d) mutagenesis of endogenous miRNA binding sites with CRISPR/Cas9, and (e) the connection of misregulation of neuronal fate to behavior. The paper could be further strengthened by addressing the following issues.

Major issues:

1. The study suggests that the primary role of ttx-1 is to repress che-1-dependent ASE marker expression in the AFDs, and that ttx-1 and che-1 may interact genetically due to co-localized expression in the AFDs. This argument could be strengthened through experiments testing whether de-repression of ASE markers in a ttx-1 mutant is due to che-1 gene activity, such as through the generation of a ttx-1; che-1 double mutant.

This is an excellent idea. We generated the *ttx-1(csu150); che-1(ot5012)* double mutant and observed the loss of all AFD (*gcy-8.1*) and ASE (*gcy-22.3* and *gcy-7.2*) marker expression. This complete loss of *gcy-8.1* expression is more severe than in the *che-1* mutant alone (Table 2, 28% staining) and suggests *ttx-1* may have some positive instructional role in initiating or maintaining AFD marker expression. We have now included this finding in the manuscript.

2. The authors discuss how the mutations in putative miRNA binding sites in the cog-1 3'UTR may influence ASE asymmetry and AFD fate by changes in cog-1 expression. To obtain further mechanistic insights, experiments assessing how the 3' UTR mutations influence reporter expression are necessary. They could help discern the regulatory function of these sites and thereby clarify how they mediate their respective phenotypes, an important point made by the authors in Discussion.

We appreciate the suggestion on how to improve the thoroughness of our work. However, stable transgenic reporter strains with extrachromosomal arrays are ~100x more difficult to obtain in *P. pacificus* than in *C. elegans* (see Methods), in part due to transgene toxicity, while still having to contend with issues of mosaicism and copy number variation. Genomic knock-in of >100 bp is also currently not technically feasible in *P. pacificus* (i.e. unable to knock in GFP or RFP). Therefore, we introduced early stop codons in the *Ppa-cog-1* reading frame to corroborate the phenotype of *cog-1(csu253)* 3' UTR mutant, which resembles a loss-of-function phenotype (i.e. loss of COG-1 protein function in the ASER leads to the 2xASEL* phenotype).

3. It is best practice to show all data mentioned in the text, rather than writing (not shown), even when the data is negative.

We have removed the 2 instances mentioning of the silent GFP reporters for *gcy-5*, *gcy-22.2*, and *gcy-22.4*.

4. The results are somewhat disorganized, making the logic hard to follow. This could be improved by addressing the following:

- The title of each section is not always reflective of all of the results within that section. For example, the title of the second results section, "gcy-8 paralogs mark AFD neurons", does not encapsulate the findings within that section that expression of these genes is dependent on CHE-1, or that the ASE rGC subfamily expression is also

dependent on CHE-1. Similarly, “Regulators of ASE left-right asymmetry” does not reflect the behavioral findings in this section.

We have rephrased the subheadings to describe all of the major findings in those sections.

- It would help with readability to organize figures in the order in which they are called in the paper, both within the figure and between figures. For example, Fig 2 panels D-H are referenced in the text before Fig 2C-F. Similarly, Fig 4 A-C are discussed before Fig 3D, 3H, and 3L. Additionally, it is necessary to call out relevant panels rather than entire figures. For example, Fig 11 is called as a whole 3 times, despite the statements referencing the figure only discussing specific panels.

We have reorganized the mentioning of Fig. 2 and Fig. 4 panels in the order they are mentioned in the text.

- The Introduction, Results, and Discussion can be shorter, more succinct. Throughout the text, the language can be improved by being more precise and less redundant. Phrases like “invisible hands of miRNA regulation” should be eliminated. The phrase “familiar guild of TFs interacting in unfamiliar ways” does not provide any substantial information.

We have removed and rephrased both passages and sought to reduce the number of words.

Minor issues:
It would be helpful to color code the first figure to match the color code used throughout the rest of the paper (AFD in purple, ASEL in red, ASER in green).

We have changed the color coding in the gene phylogeny in Figure 1 to match the color conventions used in the rest of the paper.

In the legend of Figure 3, the n= values are missing.

We have added the sample sizes.

The summary schematic of Figure 7 ignores potential contributions of the AFD neurons to behavior.

Thank you for asking why we did not speculate on the contribution of the AFD neurons in chemotaxis behavior. In *Ppa-die-1* mutants, the misexpression of *gcy-7.2* in AFD

neurons represents only 27% of J2-Adult animals compared to the mis-expression of *gcy-22.3* in ASEL neurons in 73% of animals (Table 3). Therefore, our summary schematic is based on the dominant 2xASER* phenotype.

Figure 12: It would be helpful to the reader to include a schematic of the C. elegans ASE regulatory network (bistable feedback loop) for comparison.

We have added the *C. elegans* ASE regulatory network as well as included *miR-8345/lsy-6* as a specific miRNA acting on the *cog-1* 3' UTR in the summary figure (new Figure 13).

Have all data underlying the figures and results presented in the manuscript been provided?
Large-scale datasets should be made available via a public repository as described in the *PLOS Genetics* data availability policy, and numerical data that underlies graphs or summary statistics should be provided in spreadsheet form as supporting information.

Dylan upload raw staining data!!!

Reviewer #1: None

Reviewer #2: None

Reviewer #3: Yes

Reviewer #4: Yes

PLOS authors have the option to publish the peer review history of their article (what does this mean?). If published, this will include your full peer review and any attached files.

If you choose "no", your identity will remain anonymous but your review may still be made public.

Do you want your identity to be public for this peer review? For information about this choice, including consent withdrawal, please see our Privacy Policy.

Reviewer #1: No

Reviewer #2: No

Reviewer #3: No

Reviewer #4: **Yes:** Paschalis Kratsios

Figure resubmission:

While revising your submission, please upload your figure files to the Preflight Analysis and Conversion Engine (PACE) digital diagnostic tool, https://pacev2.apexcovantage.com/. PACE helps ensure that figures meet PLOS requirements. To use PACE, you must first register as a user. Registration is free. Then, login and navigate to the UPLOAD tab, where you will find detailed instructions on how to use the tool. If you encounter any issues or have any questions when using PACE, please email PLOS at figures@plos.org. Please note that Supporting Information files do not need this step. If there are other versions of figure files still present in your submission file inventory at resubmission, please replace them with the PACE-processed versions.

Reproducibility:

To enhance the reproducibility of your results, we recommend that authors of applicable studies deposit laboratory protocols in protocols.io, where a protocol can be assigned its own identifier (DOI) such that it can be cited independently in the future. Additionally, PLOS ONE offers an option to publish peer-reviewed clinical study protocols. Read more information on sharing protocols at https://plos.org/protocols?utm_medium=editorial-email&utm_source=authorletters&utm_campaign=protocols
