# Supplementary material for: The rewiring of a terminal selector regulatory cascade generates convergent neuronal laterality
Source: PLoS Genet. 2026 Feb 11;22(2):e1011782. doi: 10.1371/journal.pgen.1011782 (PMC12919926; doi:10.1371/journal.pgen.1011782)
Supplement: Attachment — Submitted filename: Response to reviewers_second revision.docx [file pgen.1011782.s015.docx]

**Authors’ summary on the second revision of the manuscript**

We proceeded with a reviewer’s request to perform high-throughput sequencing of miRNAs in the *pash-1* reduction-of-function mutant, which is summarized in SI Figure 5. As expected from the similar “2xASER” phenotype between the *miR-8345* and *pash-1(csu227)* mutants, *pash-1* mutants show a loss of reference miRNAs and more sequence variants compared to wildtype. Thus, the *Ppa-pash-1* allele resulted in a genome-wide loss of correctly-processed mature miRNAs.

**Additional Editor Comments:**

The authors need to address the issue raised by Reviewer 1 regarding the pash-1 allele


**Reviewers' comments:**

Reviewer's Responses to Questions

**Comments to the Authors:
Please note here if the review is uploaded as an attachment.**

Reviewer #1: I thank the authors for their response to the first line of questions. I'm mostly happy but I'm afraid that I don't accept the authors' response to my point about validating that the pash-1 ts allele leads to loss of miRNAs genome-wide. Without confirming this the experiment cannot be interpreted, so I think it is very much inside the scope of the manuscript. If it is not possible to do high-throughput sequencing of small non-coding RNAs (although this is really no longer prohibitively expensive, as I pointed out in my previous review) the authors could consider northern blot analysis of several miRNAs or qPCR analysis of a panel of miRNAs.

We have performed the small RNAseq to validate that, as expected, the reduction-of-function *Ppa-pash-1(csu227)* mutant shows a global reduction of mature miRNAs that could be mapped to reference miRNAs, consistent with defects in miRNA biogenesis. A summary of the RNAseq is shown as SI Fig. 5 along with additional differential expression outputs in a compressed Supporting Information folder.

Reviewer #3: Overall, the authors improved substantially the manuscript with their revisions. It is a bit long, but not excessively so. Most of the recommendations I had were properly adressed, save for a couple:

the Ppa-pash-1 phenotype is, as expected, highly pleiotropic. I feel that the authors should mention this in the manuscript, and maybe explain why they are confident about the fact that the cells are there, but misspecified (it is not immediately obvious based on the figures). The authors may also mention that the worms are too sick to perform assays on.

We are confident that the both ASE neurons are present in the *Ppa-pash-1(csu227)* mutant because only two neurons ever stain with either *gcy-7.2 (red)* or *gcy-22.3* (green), but we never observe only a single or no *gcy-7.2 (red)* or *gcy-22.3* (green) staining as would be the case if one or both ASE neurons are altogether absent.

A quick mention of the fact that most functional comparisons were between C. elegans and C. briggsae, and how P. pacificus is much more divergent - put an estimate of divergence time, so that non-worm people can have an idea of the significance of functional conservation.

We have added the significance of our comparison with an estimate of the evolutionary divergence time between *P. pacificus* and *C. elegans* in the Introduction.

Finally, I would encourage the authors to read through very carefully to catch minute errors - eg. misformated reference, as on page 22 with Ahmed et al. without a publication year, or a few misplaced spaces.

We reformatted this citation and fine combed the entire manuscript.
